# Comparison of foot orthoses made by podiatrists, pedorthists and orthotists regarding plantar pressure reduction in The Netherlands

**DOI:** 10.1186/1471-2474-6-61

**Published:** 2005-12-20

**Authors:** Nick A Guldemond, Pieter Leffers, Nicolaas C Schaper, Antal P Sanders, Fred HM Nieman, Geert HIM Walenkamp

**Affiliations:** 1 Department of Orthopaedic Surgery, University Hospital Maastricht, The Netherlands; 2 Department of Epidemiology, University Maastricht, The Netherlands; 3 Department of Rehabilitation Medicine, University Hospital Maastricht, The Netherlands; 4 Department of Internal Medicine, University Hospital Maastricht, The Netherlands; 5 Department of Clinical Epidemiology and Medical Technology Assessment, University Hospital Maastricht, The Netherlands

## Abstract

**Background:**

There is a need for evidence of clinical effectiveness of foot orthosis therapy. This study evaluated the effect of foot orthoses made by ten podiatrists, ten pedorthists and eleven orthotists on plantar pressure and walking convenience for three patients with metatarsalgia. Aims were to assess differences and variability between and within the disciplines. The relationship between the importance of pressure reduction and the effect on peak pressure was also evaluated.

**Methods:**

Each therapist examined all three patients and was asked to rate the 'importance of pressure reduction' through a visual analogue scale. The orthoses were evaluated twice in two sessions while the patient walked on a treadmill. Plantar pressures were recorded with an in-sole measuring system. Patients scored walking convenience per orthosis. The effects of the orthoses on peak pressure reduction were calculated for the whole plantar surface of the forefoot and six regions: big toe and metatarsal one to five.

**Results:**

Within each discipline there was an extensive variation in construction of the orthoses and achieved peak pressure reductions. Pedorthists and orthotists achieved greater maximal peak pressure reductions calculated over the whole forefoot than podiatrists: 960, 1020 and 750 kPa, respectively (p < .001). This was also true for the effect in the regions with the highest baseline peak pressures and walking convenience rated by patients A and B. There was a weak relationship between the 'importance of pressure reduction' and the achieved pressure reduction for orthotists, but no relationship for podiatrists and pedorthotists.

**Conclusion:**

The large variation for various aspects of foot orthoses therapy raises questions about a consistent use of concepts for pressures management within the professional groups.

## Background

A variety of foot impairments such as rheumatoid arthritis and diabetes are associated with elevated plantar forefoot peak pressures and metatarsalgia[1]. Reduction of forefoot plantar pressure through foot orthoses is a common treatment for these conditions and for non-specific metatarsalgia. Moreover, forefoot pain is one of the most common foot complaints for which foot orthoses are prescribed[2]. Several studies have shown effectiveness of foot orthoses and/or inserts on pressure reduction in patients with metatarsalgia [3-10], but there seems to be considerable variation for different aspects of orthoses therapy such as prescription habits [11-14], foot examination [15-22]and casting[23]. No publications on the evaluation of different professional groups are available as far as plantar pressure management is concerned.

Dutch medical specialists and general practitioners prescribe foot orthoses which are mainly provided by podiatrists, pedorthists (orthopaedic shoemaker) and orthotists (orthopaedic technician). These disciplines have separate vocational training. They differ regarding diagnostic procedures, construction of orthoses and therapeutic approach e.g.: pedorthists are specialized in foot orthosis therapy for orthopaedic shoe wear, whereas podiatrists and orthotists mainly provide foot orthoses for non-orthopaedic shoe wear. In general, orthotists take care of more severe disorders than podiatrists[14]. Although each discipline has a specific focus on particular foot problems, all three disciplines provide foot orthoses for comfort shoes to treat foot impairments associated with elevated plantar forefoot peak pressures: e.g. higher than 700 kPa[24,25]. It is not known what the consequences of these differences are for the extent of pressure reduction. The purpose of this study was to evaluate the effect of custom-made foot orthoses, made by representatives of these professional groups in the Netherlands, on forefoot plantar pressure and walking convenience. Three patients with metatarsalgia and elevated forefoot plantar pressure were chosen as examples to show the effects of the orthoses. The specific aims were to assess possible differences in plantar pressure reduction in these patients between the professional groups, its variability within the groups and the differences of that variability between the three groups. In addition, the relationship between the importance of pressure reduction as stated by the therapist and the effect on plantar peak pressure through foot orthoses was evaluated.

## Methods

### Therapists

As representatives of their professional groups, ten podiatrists, ten pedorthists and eleven orthotists from the southern part of The Netherlands were asked to construct foot orthoses for three patients with metatarsalgia and elevated forefoot peak pressure. Podiatrists were approached through telephone directories. Companies of pedorthists and orthotic workshops were approached through member lists of the professional associations. Each delegated between one and three therapists for the study. The median professional experience in years was 7.5; 16.5 and 20, respectively for podiatrists, pedorthists and orthotists.

### Patients

Three patients, 2 females of 60 and 61 years old and a 37 years old male, with forefoot complaints, elevated plantar peak pressures and an indication for foot orthoses were selected from an orthopaedic outpatient clinic. The male patient had foot problems related to psoriatic arthritis, which was inactive during the study period. The specific diagnosis for both female patients was bilateral metatarsalgia due to overloading of the forefoot as consequence of structural defects (table 1) leading to functional anomalies. Additional details about the patients are given in table 1. Before the start of the study, patients were informed about all study procedures and their possible risks. The Research Ethical Committee of the University Hospital Maastricht approved the study.

**Table 1:** Patient characteristics

Patient	**A**		**B**		**C**	
Gender	female		female		male	
Age (yr)	60		61		37	
Weight (kg)	105		73		82	
Body length (cm)	178		154		181	
Preferred walking speed (m.s^-1^)	1,63		0,88		1,72	
Systemic diseases					Arthritis psoriatica	
	left	right	left	right	left	right
**Structural classification**						
pes plano valgus	**x**	**x**				
calcaneus valgus					**x**	**x**
hallux valgus			**x**	**x**		
bunion			**x**	**x**		
claw toes					**x**	**x**
**Specific diagnosis**						
Metatarsalgia	**x**	**x**	**x**	**x**	**x**	**x**
Plantar fasciitis		**x**				
MTP-1 joint Extension	50°	50°	50°	35°	55°	60°
MTP-1 joint Flexion	45°	40°	30°	35°	400	45°
*Bare foot peak pressure (kPa)	907	506	771	662	1202	826

Yr = years, kg = kilogram, cm = centimetre, m = metre, s = second,

MTP = metatarsophalangeal, *highest value under the plantar forefoot is showed.

### Casting and construction of foot orthoses

Two sessions were organised at the University Hospital Maastricht with an interval of three weeks between sessions. The first session included patient examination and casting. Therapists were asked to rate the 'importance of pressure reduction' per patient through line bisection of a 100 millimetre visual analogue scale. The orthoses were constructed in the three weeks between the sessions. During the second session, before final delivery, the therapists had an opportunity to check the constructed orthoses for adequacy. If deemed necessary, accommodations of orthoses were made.

### Inshoe plantar pressure measurement and evaluation of foot orthoses

Per patient 31 pairs of foot orthoses were evaluated twice in two sessions. Two pairs of sham orthoses were added to create eleven blocks of three pair of orthoses, one from each discipline. The blocks were randomized to determine the measurement sequence and this sequence was reversed for the second session. All orthoses were evaluated in the patient's own shoes and wearing standard thin socks while walking on a treadmill. The Pedar Insole-system^® ^(Novel, Munich) was used to measure plantar pressures[26,27]. The first author performed all measurements. Whether these off the shelf shoes had enough space for adding orthoses and the Pedar insole was checked. Patients individually chose their preferred walking speed (table 1), which was kept the same for all subsequent measurements. To minimize a possible effect of the test sequence, such as a possible effect of fatigue on plantar pressures, an equal number of steps from both sessions were used for evaluation. Data were recorded for approximately 50 seconds with a frequency of 50 frames per second. Plantar peak pressure was estimated by calculating the mean over the readings of 40 steps (20 steps per session). This was done for six separate regions: big toe (BT) and metatarsal one (mt-1) to five (mt-5) through anterior-posterior radiographs and Novel 'create any mask^®^' software[28]. Baseline values of inshoe plantar pressures, i.e. without orthoses, were calculated as the mean of 4 measurements: for each session, one before and one at the end of the test series. In order to check the influence of fatigue on peak pressure, we compared the before and after values without orthoses of each test session. After each measurement, patients scored walking convenience for left and right orthosis on a ten-point scale on a questionnaire: How do you rate the walking convenience of these orthoses (0 = extremely bad, 10 = excellent)? Walking convenience was estimated by calculating the mean score of the two sessions.

### Analysis

Differences in plantar pressure reduction amongst the professional groups were evaluated for the region with the highest peak pressure measured under the forefoot without orthosis (table 2). For patient B and C the effect on peak pressure was also evaluated for their right and left BT regions respectively, because pressures in these regions were high compared to the peak pressures under the metatarsals. At first plantar pressure data were investigated for normal distribution by the Kolmogorov-Smirnov test. If the data did not meet the assumptions for parametric analysis, a Friedman test was used for non-parametric overall analysis. If there was a statistically significant difference between the groups, Wilcoxon Signed Ranks Tests were performed for pair-wise comparison. We also evaluated the effect of the orthoses focusing on the region were the peak pressure reduction was maximal. For this, the difference between the peak pressure measured with orthoses and the baseline value was calculated for each forefoot region. The largest difference was used for this evaluation.

**Table 2:** Baseline inshoe peak pressures (kiloPascal).

	**A left**	**A right**	**B left**	**B right**	**C left**	**C right**
**BT**	208	200	204	**298**	**466**	200
**mt-1**	359	**378**	250	258	234	147
**mt-2**	**429**	358	218	249	292	291
**mt-3**	428	270	**332**	**280**	**336**	**332**
**mt-4**	237	226	266	219	207	307
**mt-5**	214	173	200	174	69	200

Highest baseline inshoe peak pressures for mt regions are bold printed. For patient B & C, BT regions were added because of high local peak pressures.

BT = big toe, mt = metatarsal

Variance of the maximal pressure reduction was used to show the within-group variation and to test for equality of these within group variations between the three disciplines. For this purpose a homogeneity of variance analysis was performed with a repeated measures ANOVA, using the GENOVA computer program[29].

For the assessment of the relationship between achieved pressure reduction and 'importance of pressure reduction' we used the maximal pressure reduction. For this purpose linear regression analysis was performed for the cases were there was a wide distribution of the 'importance of pressure reduction' ratings. Statistical analysis was carried out with SPSS 12.01^® ^software (SPSS Inc.). An alpha level of 0.05 was chosen to judge statistical significance.

## Results

### Description of constructed orthoses

Orthoses made by podiatrists clearly differ from orthoses from pedorthists and orthotists. All podiatrists constructed thin insoles out of rubber, leather and cork, but they varied in the application of corrective and/or supportive elements. In broad outlines, orthoses of pedorthists and orthotists were similar. The pedorthists and orthotists made orthoses that look more like the 'Root style' orthoses[14,30]. However, within the professional groups, there was a considerable variation in applied materials and the use of corrective and supportive adaptations. All orthoses from podiatrists were fully custom-made while this was only the case for 56% of orthoses from pedorthists and 45% of those from orthotists. The remainder were partly or completely constructed from prefabricated elements. Podiatrists constructed full-length orthoses for all patients. Pedorthists provided 34% full-length orthoses, 20% three-quarter and 46% 'in between' length orthoses. Orthotists provided 58% full-length orthoses, 24% three-quarter and 18% 'in between' length orthoses.

### Plantar pressure measurements

For all three patients, the changes of the plantar pressure measurements without orthoses, from before to after the test series, were very small. The change during session 1 for patient A was 0.4, patient B -4.5, patient C 5.1 kPa. During session 2: patient A -0.3, patient B -4.3, patient C -2.1 kPa. We concluded that there was no relevant effect of fatigue on peak pressures in the course of the measurement sessions.

### Effect of orthoses on highest pressure regions for patient A

**Figure 1:**
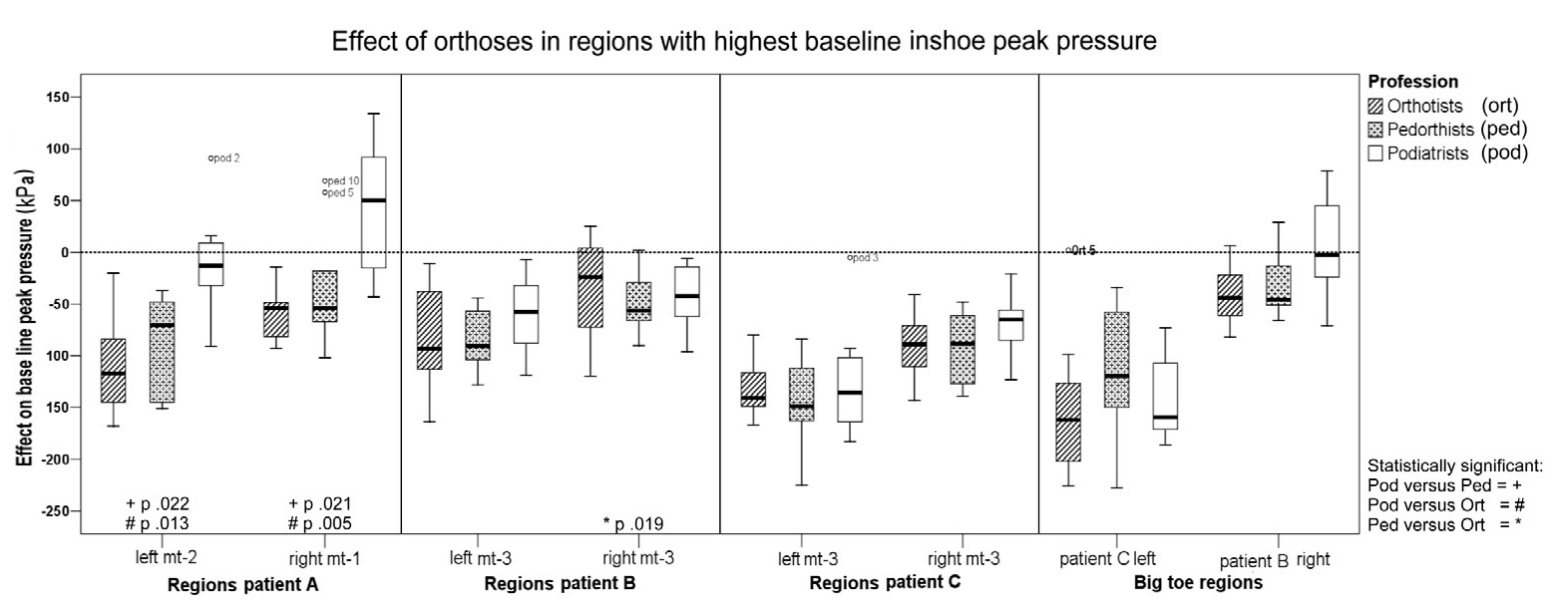
**The effect of orthoses in regions with the highest baseline inshoe peak pressure**. Boxplots show the median, interquartile range, outliers (o), and extreme cases (*) of individual variables.

Results for each region for pair wise statistical testing are listed in the figure 1. The highest baseline peak pressures for patient A were measured in the left mt-2 and the right mt-1 regions (table 2). With respect to the left mt-2 region, orthotists and pedorthists achieved greater median reductions, 117 kPa and 71 kPa, than podiatrists, 13 kPa: p = .022. For the right mt-1 region, both orthotists and pedorthists achieved a greater median reduction of 54 kPa, while the orthoses of podiatrists resulted in an increase of peak pressure by 50 kPa. The differences between orthotists and pedorthists compared to podiatrists were statistically significant: p = .021.

### Effect of orthoses on highest pressure regions for patient B

The highest baseline peak pressures for patient B were measured in both mt-3 regions (table 2). Orthotists, pedorthists, and podiatrists achieved median reductions of 93, 91 and 58 kPa respectively: figure 1. For the right mt-3 region, these reductions were 57, 24 and 43 kPa respectively. The difference between orthotists and pedorthists was statistically significant: p = .019 (figure 1).

### Effect of orthoses on highest pressure regions for patient C

The highest baseline peak pressures for patient C were also measured in both mt-3 regions (table 2). Orthoses of pedorthists, orthotists and podiatrists achieved a median reduction of 149, 141 and 136 kPa respectively in the left mt-3 region (figure 1). For the right mt-3 region, these reductions were 89, 83 and 65 kPa respectively (figure 1).

### Effect of orthoses on highest pressure in big toe regions of patient B and C

For patient B and C, a high peak pressure was measured in the corresponding right and left BT region (table 2). Orthoses of pedorthists, orthotists and podiatrists achieved a median reduction of 46, 44 and 3 kPa respectively, in the right BT region of patient B (figure 1). For the left BT region of patient C, orthoses of orthotists, podiatrists and pedorthists achieved a median reduction of respectively 162, 160 and 120 kPa (figure 1).

### Within group variances of maximal peak pressure reduction

The maximal reduction of peak pressure calculated for the whole plantar surface over all forefeet was greater with orthoses of pedorthists (p = .005) and orthotists (p < .001) than orthoses of podiatrists: -96 and -102 versus -75 kPa respectively. The within-group variability, expressed as standard deviation, of the maximal local peak pressure reduction for the whole forefoot were 16.0, 14.2 and 16.3 kPa for podiatrists, pedorthists and orthotists respectively (table 3). The differences of the within-group variances between the disciplines were not statistically significant.

**Table 3:** Maximal peak pressure reduction calculated for the total plantar surface of all forefeet (kiloPascal).

	**Mean reduction**	**SD**	**Minimum-Maximum**
**Podiatrists**	-75	16.0	-186 : 4
**Pedorthists**	-96	14.2	-226 : -15
**Orthotists**	-102	16.3	-228 : -18

Δ = change, SD = standard deviation

podiatrists versus pedorthists: p = 0.244

podiatrists versus orthotists: p = 0.131

pedorthists versus orthotists: p = 0.261

### The relationship between the 'importance of pressure reduction' and the effect on peak pressure

**Figure 2:**
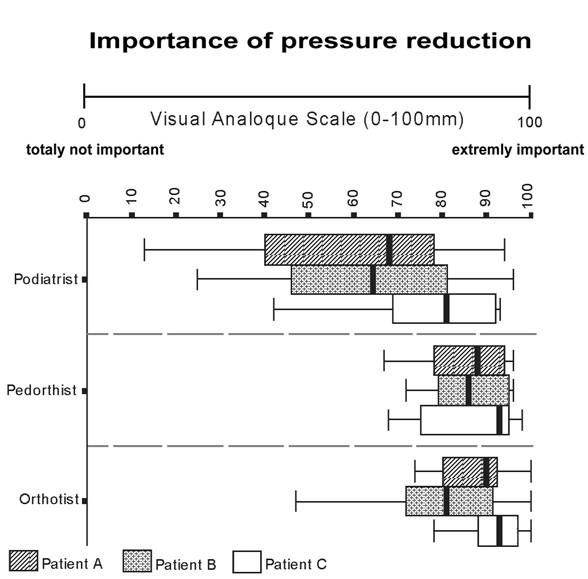
**Importance of pressure reduction (quartiles)**. Zero millimetre indicates totally not important and 100 millimetre indicates extremely important. Boxplots show the median and interquartile range.

Box and whisker plots show the median scores and quartiles for 'importance of pressure reduction' rated by the therapists (figure 2). The median ratings by podiatrists for patients A, B and C were respectively: 68, 65 and 81 out of 100 mm. The ratings by pedorthists for patients A, B and C were 88, 86 and 93 mm, respectively. The ratings by orthotists for patients A, B and C were 90, 81 and 93 mm respectively.

**Figure 3:**
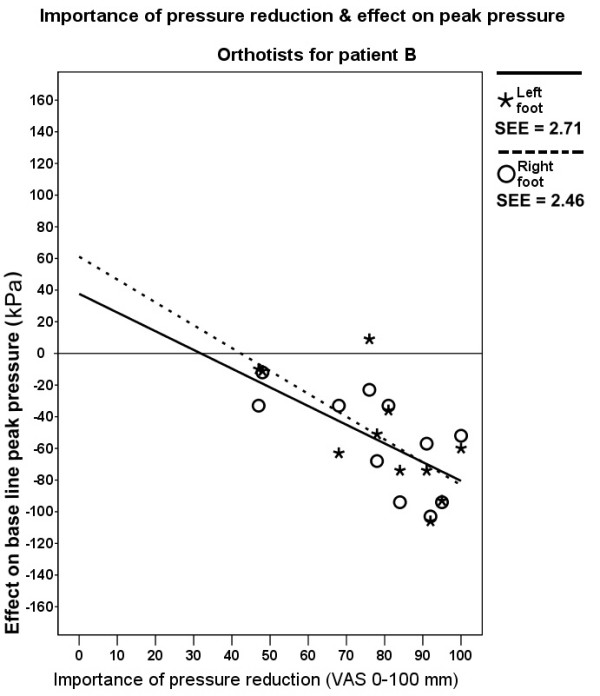
**Importance of pressure reduction rated by orthotists for patient B & effect on peak pressure**. SEE = standard error of the estimate.

The relationship between the 'importance of pressure reduction' and the effect on peak pressure for the whole forefoot was only evaluated for patients for which therapists within professional groups had a large variation in their judgement of the importance of pressure reduction: for podiatrists patients A and B and for orthotists patient B (figure 3).

Linear regression analysis showed only statistically significant weak negative associations between the 'importance of pressure reduction' ratings by orthotists and the effect on peak pressure for the left and right foot of patient B. Importance of pressure reduction explained 50% (p = .015) and 48% (p = .019) of the variance of the effect on peak pressure, respectively (figure 3). There was no statistically significant relation between ratings of podiatrists and effect on peak pressure for patient A and B.

Podiatrists had less years of professional experience than pedorthists (p = .037) and orthotists (p = .008), while orthotists and pedorthists did not differ in years professional experience. Multiple regression analysis showed that there was no, or a very weak, relationship between achieved pressure reduction and years professional experience, number of constructed orthoses per week, or importance of pressure reduction (data not shown).

The appreciation of walking convenience by the patients is shown in table 4 (Means were calculated through the mean of two ratings per therapist). Walking convenience was better rated by patients A and B for orthoses of pedorthists and orthotists compared to orthoses of podiatrists. The differences for both feet of patient B were statistically significant (left: p = .005, right: p = .005). For patient A, only the differences between orthotists and podiatrists for both feet were statistically significant (left: p = .019, right: p = .047). According to the ratings of patient C, pedorthists scored, on average, better than podiatrists and podiatrists better than orthotists.

**Table 4:** Mean effects of orthoses on walking convenience (10-point scale)

	**Patient A**		**Patient B**		**Patient C**	
	**Left**	**Right**	**Left**	**Right**	**Left**	**Right**
**Podiatrist**	6.5 ± 0.3	6.5 ± 0.6	4.0 ± 0.4	4.0 ± 0.4	4.8 ± 1.3	5.0 ± 0.9
**Pedorthist**	7.5 ± 1.1	7.5 ± 0.8	7.0 ± 1.0	7.0 ± 1.0	5.0 ± 1.6	5.3 ± 1.3
**Orthotist**	7.5 ± 0.8	7.5 ± 0.7	6.5 ± 1.0	6.5 ± 1.0	4.5 ± 1.3	4.5 ± 1.3

Walking convenience scale 0–10 points (0 = extremely bad, 10 = excellent). Means were calculated through the mean of two ratings per therapist, (± ...) = standard deviation.

## Discussion

We studied the effect of 186 custom-made foot orthoses (31 therapists, 3 patients, 2 feet) made by podiatrists, pedorthists and orthotists on plantar pressure and walking convenience for three patients with elevated forefoot peak pressures and metatarsalgia. Within the three disciplines there was a large variation in construction of the orthoses and achieved peak pressure reductions. On average, the pedorthists and orthotists achieved slightly greater pressure reductions and a better walking convenience than podiatrists.

In accordance with what was reported in other studies, it turned out to be impossible to categorize the constructed orthoses in general orthotic classifications, such as 'functional' or 'accommodative'[11]. Therefore, we only described aspects of the shape of the orthoses and the materials used. Generally speaking, the orthoses made by pedorthists and orthotists were similar to orthoses of Anglo-Saxon podiatrists, although, rigorous application of procedures for casting and construction of functional orthoses according to the American College of Foot & Ankle Orthopaedics & Medicine (ACFAOM) and the Australian Podiatry Council practice guidelines[31,32], were not commonly used. Orthoses of podiatrists were constructed according to the principles described by Lelièvre[33] and further developed by Lavigne and associates [34-36]. They were based on pressure sheet and physical examination and constructed as a thin sole with delicate corrective and/or supportive elements made from leather, rubber and cork. The Dutch podiatric orthoses are different from the custom-made foot orthoses made by the Anglo-Saxon podiatrists, which are usually constructed according to the concepts formulated by Root et al[11,16,30,37,38].

We assessed the pressure reduction effect of orthoses for the region with the largest peak pressure. This is where the largest effects should be achieved. In general, pedorthists and orthotists achieve greater reductions, than podiatrists. However, these differences in peak pressure reductions between podiatrists versus pedorthists and orthotists were statistically significant for only one of the patients. In addition, the maximal local reduction of peak pressure calculated for the whole plantar surface over all forefeet was statistically significantly greater with orthoses of pedorthists and orthotists than orthoses of podiatrists.

In one of the patients, at the location of the highest baseline peak pressure, the podiatric orthoses even resulted in a increase of the peak pressure. Such an increase of plantar pressure could be dangerous, especially in neuropathic feet[39,40].

According to the ratings for 'importance of pressure reduction', optimizing pressure distribution was a main treatment goal for most therapists (figure 2). However, there was no relationship for podiatrists and a weak relationship for orthotists (figure 3) between the supposed 'importance of pressure reduction' and the achieved pressure reduction. This implies that there is a discrepancy between treatment goal and treatment effect with respect to plantar pressure reduction. This is worrisome because optimizing pressure distribution is an important aim of foot orthoses therapy [25].

Walking convenience is important for the patient's preference and acceptance of an orthosis for daily use. Two patients scored walking convenience better for orthoses of pedorthists and orthotists compared to orthoses of podiatrists. For patient C there were no clear differences. However, because walking convenience was judged immediately after the measurement session, it remains unclear whether the patient's preference will stay the same after getting used to the orthosis.

The general theoretical and practical concepts of foot orthoses therapy should be common to the therapists of each discipline concerned. The situation in the Netherlands for establishing this professional uniformity is optimal because there is only one school for each discipline. Nevertheless, there is a large variation in orthoses construction and treatment effects within each discipline, while there were no differences in the variation in effect on peak pressure among the three disciplines.

We evaluated the effects of foot orthoses therapy on plantar pressure and walking convenience for only three patients. We did not set out to examine patient-specific therapy outcome. Although the effects of orthoses for patients with other foot pathologies and plantar pressure patterns could be different, we see no reasons why the differences between the professional groups and the variation within professional groups would be substantially different for other patients. We are of the opinion that the therapists who participated in our study are a fair representation of their national colleagues. There could even be some underestimation of the variation within professional groups because some of the therapists work within the same company.

Variation in plantar pressure management could be the result of inconsistent application of diagnostic procedures, of setting treatment goals and methods of constructing orthoses. In a previous study we indeed showed that therapists often disagree about the locations with high plantar pressures[41]. Information about variability of clinical achievements among therapists is essential for the interpretation of the results of studies where foot orthoses therapy is evaluated[42,43]. Only when there is a small variation is it possible to extrapolate the effect of therapy achieved by one therapist to his or her colleagues.

We showed a large variation among therapists for the Dutch situation. It is difficult to say whether these results can be generalised to professional groups in other countries. However, we do not know of any study showing that the situation is better elsewhere. Insofar as variation has been studied, specific aspects like diagnostic procedures and orthoses prescription show similar variability problems[11,23,44-52].

## Conclusion

This study showed differences between three disciplines that construct custom foot orthoses for patients in The Netherlands with respect to construction, presumed importance of plantar pressure reduction, achieved pressure reduction and walking convenience. On average, pedorthists and orthotists achieved a slightly larger pressure reduction in high peak pressure regions and a better walking convenience than podiatrists did. There was a weak relationship between the 'importance of pressure reduction' and the achieved pressure reduction for orthotists, but no relationship for podiatrists and orthotists. The large variation in construction of the orthoses and in peak pressure reduction within the professional groups raises questions about a consistent application of therapeutic concepts for the management of elevated peak pressures.

A better understanding of and consensus about the mechanisms underpinning the effectiveness of foot orthoses therapy is needed in order to improve foot orthoses therapy, This must lead to unambiguous guidelines that enable improved education and consequently less variation between therapists.

## Competing interests

The author(s) declare that they have no competing interests.

## Authors' contributions

All authors designed the study. NG collected and prepared the data. NG, PL and FN analyzed the data. All authors interpreted and discussed the data. NG, PL and GW drafted the manuscript. NA and PL revised the manuscript. All authors read and approved the final manuscript.

## Pre-publication history

The pre-publication history for this paper can be accessed here:


